# DBT-PAHSE Intervention for Reduce Emotion Dysregulation and Suicide Behavior in Mexican Early Adolescents: A Longitudinal Study

**DOI:** 10.3390/healthcare11091311

**Published:** 2023-05-03

**Authors:** Alicia E. Hermosillo-de-la-Torre, Stephania M. Arteaga-de-Luna, Paulina Arenas-Landgrave, Catalina González-Forteza, Denise L. Acevedo-Rojas, Kalina I. Martínez, María E. Rivera-Heredia

**Affiliations:** 1 Psychology Department, Center for Social Sciences and Humanities of The Autonomous University of Aguascalientes, Aguascalientes 20100, Mexicokalinamartinez@hotmail.com (K.I.M.); 2 Faculty of Psychology, National Autonomous University of Mexico, Mexico City 04510, Mexico; palandgrave@gmail.com; 3 National Psychiatry Institute, Psychosocial Studies, Mexico City 14370, Mexico; catiartes@gmail.com; 4 Psychology Faculty, Michoacan University of San Nicolás de Hidalgo, Morelia 58110, Mexico; maria.elena.rivera@umich.mx

**Keywords:** DBT skills, suicide prevention, adolescents, emotional dysregulation, self-injuries, suicide thinking

## Abstract

This study aimed to evaluate the effect of time on suicidal behavior, associated risk factors, and protective factors in early Mexican adolescents. Method: With a two-year longitudinal design, which included 18 of 34 adolescents who had previously participated in a DBT skills training program (DBT-PAHSE). The study evaluated ideation, suicide attempt, depression, emotional dysregulation, and psychological resources. Results: We observed differentiating significant differences over time in emotional dysregulation (F = 2.36 *p* = 0.04, η^2^= 0.12, β = 0.72), affective resources (F = 3.94, *p* = 0.01, η^2^ = 0.18, β = 0.82), and suicidal ideation. (F = 2.55, *p* = 0.03, η^2^= 0.13, β = 0.77). In conclusion, the DBT-PAHSE program prevented deaths by suicide. It showed a reduction in emotional dysregulation up to two years after the end of treatment and maintained an increase in emotional and social resources. However, improvements are required to reduce depression over time and strengthen psychological resources.

## 1. Introduction

Adolescence is a life cycle stage characterized by biological, social, and psychological changes intrinsically associated with mental health and psychosocial well-being [1,2]. The periods ranging from 10 to 12 and 13 to 15 years, known respectively as “early adolescence” and “middle adolescence” [3], are critical to the development and maintenance of healthy behaviors such as [4] acquiring adequate sleep [5], exercise and eating habits [6], as well as learning skills to establish healthy interpersonal relationships, solve problems and manage emotions. However, this is also a critical period to identify mental disorders [2] timely and understand the trajectories of suicidal risk behaviors that will appear during late adolescence and adulthood [7].

During early and middle adolescence, the observed increase in deaths by suicide is a disturbing problem. According to the Pan American Health Organization [8], in the countries that make up the Region of the Americas, mortality from this cause increased considerably in people aged 10 to 24 from 2010 to 2014. In Mexico, the percentage of the relative change of deaths by suicide in this population group has shown an increase of 415% in the last two decades [9]. PAHO data [10] indicate that the increase has [11] been 86% in the last ten years, with a greater magnitude in women (155%) than in men (61%). Suicide attempts have also increased considerably in this age group. A comparative analysis of prevalence ratios for lifetime suicide attempts among Mexican adolescents showed a 300% relative increase in the risk of having a suicide attempt during the year analyzed [12]. The results also showed that the groups with the sharp increases were women and adolescents from 10 to 12 years of age.

The development and appearance of suicidal behavior, as well as the increased risk of suicide observed in early and middle adolescence, is due to multiple biopsychosocial factors that interact in a complex way during infancy and childhood [13]. Among the main psychosocial risk factors associated with suicidal behavior are early experiences of adverse life events, negative parenting styles, the acquisition of impulsive and aggressive personality traits, negative affective states, and biases in cognitive processing. In addition, substance abuse and significant symptoms of anxiety and depression [14,15]. A recent study with Mexican adolescents found risk associations between suicide attempts and symptoms of depression, anxiety, hopelessness, alcohol and tobacco abuse, low self-esteem, and experiences of childhood trauma [16]. In early-onset adolescents, associations have been established between thoughts of death and desires to take one’s own life with emotions of sadness, pain, anguish, and anxiety [17].

In various regions of the world, there have been significant increases in the rates of death by suicide in this age group in the years before the COVID-19 pandemic. The conditions of social isolation adopted in said health contingency, as well as the loss of loved ones and situations of economic instability, will worsen this situation [16]; however, this is not very clear because while some countries report increases in deaths by suicide after the pandemic [18], others have not observed it. However, the prevalence of suicide attempts by children and adolescents in emergency hospital centers has increased in recent years during and after the pandemic in many regions of the world [19].

The psychosocial factors associated with the risk of suicide have also presented alarming increases in adolescence. In 2019, this group’s global prevalence of mental disorders was 14% [20]. The mental health conditions that contribute the most to this percentage are anxiety disorders (3.64%), behavioral disorders (3.56%), and attention deficit hyperactivity disorder (3.12%). Is followed by depressive disorders (1.6%), major depressive episodes (0.81%), bipolar disorder (0.18%), and finally, eating disorders (0.11%).

Given the need to reduce the suicide mortality rate, the prevalence of suicidal behaviors, and the psychosocial factors associated with them, it is necessary to have early-onset mental health interventions for adolescents. Interventions based on scientific evidence, low cost, accessible for community implementation, and focused on reducing the risk of suicide and promoting psychosocial well-being are required. In this sense, intervention strategies for learning emotional regulation skills are widely recommended [21].

Dialectical Behavior Therapy (DBT) is considered a comprehensive treatment and recognized as a transdiagnostic psychotherapeutic strategy for treating a wide range of emotional dysregulation difficulties addressed by multiple intervention components [22]. It has extensive evidence of its efficacy in treating children and adolescents with suicidal behavior, depression, eating disorders, impulsivity, and aggressiveness [23].

From the conceptual framework of DBT, emotional dysregulation (ED) is understood as the lack of ability to modify or modulate emotional cues, experiences, acts, and verbal and/or non-verbal responses to adapt them to the particular social context. This constitutes an emotional vulnerability when dysregulation becomes generalized. Generalized emotional dysregulation is characterized by experiencing a high sensitivity to emotional stimuli, immediately triggering equally intense responses with prolonged activation and a slow return to baseline. People with this type of emotional vulnerability experience a large amount of aversive emotional stimuli, inability to regulate the intense physiological arousal triggered, difficulty redirecting attention, cognitive distortions and biases in information processing, poor impulse control, difficulty organizing and coordinating activities to achieve goals and objectives, tendency to become paralyzed or dissociate under high-stress conditions [4].

One of the components of the treatment, DBT skills, has shown that it can be implemented in other non-clinical community settings such as schools [24,25,26,27]. The DBT skills component comprises four blocks: (a) mindfulness skills; (b) skills to tolerate distress, (c) abilities to regulate emotions, and (d) skills to establish healthy interpersonal relationships. According to Linehan [4], mindfulness skills are at the core of DBT skills. These skills focus on the practice of observation, description, spontaneous participation, non-judgmental nation, the focus on awareness in the present moment, and the orientation to do what is most effective instead of what is considered correct. The distress tolerance skills block represents an extension of mindfulness skills. These skills develop the ability to observe and experience unpleasant emotions without evaluating, modifying, or controlling the emotional experience. Emotional regulation skills strengthen the ability to change unpleasant emotions and cause suffering. Finally, healthy relationship-building skills promote proactive strategies for asking for what is needed, refusing to do something, and establishing mutually respectful interactions.

Cultural adaptations of evidence-based treatments are potentially beneficial, particularly for low- and middle-income countries, but have received little attention [28]. Although there are contradictory studies that question the results obtained with culturally adapted evidence-based treatments [29], there is contrary evidence that shows the need to carry out cultural adaptations of these treatments [30]. In the case of DBT, a recent study carried out by Ramaiya et al. [28] to adapt the DBT skills group to the rural population of India showed optimal results in the regulation of emotions with a program that considers both the fidelity of the DBT principles and the cultural values and norms of the population.

Despite the existing evidence on the benefits of the DBT skills learning program in reducing emotional dysregulation and suicide risk in adolescents, this evidence is still incipient in Mexico. A couple of studies have tested the effectiveness of the DBT STEPS-A and DBT-A programs with university students, showing positive effects in reducing emotional dysregulation [31,32]. Regarding evaluating the DBT skills program in adolescents from 10 to 12 years of age in Mexico, only one study was identified in which an adapted version of the DBT skills in early-onset adolescents was developed and evaluated [33]. The program called DBT-PAHSE (Socio-emotional Skills Learning Program) reported on the philosophical, theoretical, and methodological principles of Dialectical Behavioral Therapy (DBT) reported on the DBT-SKILLS [34] and DBT STEPS-A program [26]. DBT-PAHSE was implemented through a pilot study and clinical trial from 2017 to 2019; specifically, the clinical trial results suggest that it is a learning program for socio-emotional skills for suicide prevention in Mexican pre-adolescents. Well, 80% of the participants in the control group were located below the average observed in the experimental group concerning risk factors such as depressive symptoms and above in terms of self-esteem and psychological resources.

Based on the above, the present study sought to evaluate the effect of time on suicidal behavior, psychosocial risks, and associated protective factors in a group of early-onset adolescents who received a DBT skills training program (DBT-PAHSE). Secondarily, we sought to analyze whether emotional dysregulation was reduced and, with it, the risk of suicide and associated psychosocial factors during middle adolescence. In particular, the interest focused on analyzing the trajectories of emotional dysregulation, significant depressive distress, and psychological resources.

## 2. Materials and Methods

A longitudinal design study evaluates the effects of the DBT-PAHSE program (Socio-emotional Skills Learning Program informed in DBT) two years after its implementation with early Mexican adolescents. The design included a case study approach (N = 1). We took the intervention DBT-PAHSE group that participated in previous study [24]. The sequence of observations included in the period studied was as follows:

O1  TX  O2  O3  O4  O5  O6

O1 Pre-intervention evaluation

O2 Post-intervention evaluation

O3 Six months follow evaluation

O4 Twelve months follow evaluation

O5 Eighteen months follow evaluation

O6 Twenty-four months follow evaluation

### 2.1. Participants

Thirty-four adolescents chosen by convenience method participated in this study. Participants were 11 to 14 years (M = 13.78, SD = 0.428), all women because the school does not admit boys. The adolescents were originally from a municipality in the northwestern region of Jalisco, Mexico, belonging to a medium-low socioeconomic stratum. The participants were in the first year of primary and secondary education in the Mexican public system schools, all from the same school and class. Most of the participants (70%) lived with their nuclear family, 15% in an extended family, 3% in a mixed family, 9% in a single-parent family with the mother as the head of the family, and the rest in a single-family parent with father as head of the family (3%).

### 2.2. Instruments

1. The battery of Factors Involved in the Care of Life in Children and Adolescents (FICVIDA) was expressly integrated by the research team and collaborators for the evaluation. The battery settled for the following measurement instruments:

1.1. Scale of Epidemiological Studies of Depression Revised (CES-DR) developed by Randolph in 1977, adapted to Mexico by González-Forteza, Jiménez-Tapia, Ramos, and Wagner [35]. The scale has 35 five-point Likert-type items ranging from “0 days” to “8 to 14 days” and which addresses criteria related to the depressive symptomatology of the DSM-IV Major Depressive Episode present during the last two weeks. The scale obtained an Alpha index of 0.892 (Previous study alpha = 0.890).

1.2. Roberts Suicidal Ideation Scale [36]. It Assesses the presence of suicidal ideation in the last two weeks through 3 five-point Likert-type items ranging from “0 days” to “8 to 14 days”. It has an internal consistency of 0.960 (Previous study alpha = 0.875).

1.3. Suicidal Behavior Questionnaire, developed by González-Forteza, Alvares-Ruiz, Saldaña-Hernández, García, Chávez-Hérnandez, Pérez [37]. This study took only seven items: “Have you ever hurt, cut, poisoned, or hurt yourself on purpose to die?” (“yes” or “no”, response). One open question explores the age of the last time of self-injury. A four-point Likert-type item explores the number of times that he or she harmed himself (from “once” to “more than 5”). Additionally, assessed cognitive lethality through a 3-point Likert-type item that goes from “Sure, I knew I would die” to “Impossible, I knew I would not die” and assess consequences regarding emotional support or medical care through another item on a five-point Likert scale that ranges from “they took me to a hospital (emergency room)” to “there were no consequences”.

1.4. The scale of Difficulties in Emotional Regulation, created by Gratz and Roemer [38] and adapted by Marín, Robles, González-Forteza, and Andrade [39] for Mexican adolescents. The Mexican adaptation considers the acceptance of emotions, goals, impulses, conscience, regulation strategies, and clarity of emotions. It has 24 Likert-type items, five points that go from “rarely (0–10%)” to “almost always (91–100%)”. The Alfa index was 0.89 (Previous study alpha = 0.883).

1.5. Psychological Resource Scales. Developed by Rivera-Heredia, Andrade, and Figueroa [40]. That ass: (a) Affective Resources are management that the person has of his emotions, control, expression, and regulation. The scale includes self-control, anger management, and recovery of emotional balance; (b) Cognitive resources are perceptions and beliefs that they have regarding the problems that surround them, based explicitly on religious beliefs. (c) Social Resources are capabilities that individuals have to bond with others establishing relationships with permanent containment and support; it also involves asking for help when needed. Made up of the factors: Support Network, Ability to Request Support, and Altruism. Through 40 four-point Likert-style items ranging from “almost always” to “rarely.” The Alfa index was 0.89 (Previous study alpha = 0.898).

2. Digital records on the use of skills during the following six months applied through Google Forms. Record the use of the skills applied during the last month, explain the skills, and offer eight response options: “(a) I did not think about it, nor did I use it; (b) I thought about it, I did not use it because I did not want to use it; (c) I thought about it, I did not use it, but I did want to use it; (d) I tried, but could not use it; (e) I tried, I could do it, but it did not help me; (f) I tried it, I was able to use it, and it helped me; (g) I automatically used it, and it did not help me; (h) I automatically used it, and it helped me”.

### 2.3. Procedure

The present study was implemented after the application of the DBT PAHSE program, which is a universal and selective intervention implemented in educational contexts and developed under DBT philosophical, theoretical, and methodological principles, specifically on the DBT-SKILLS [34] and DBT-STEPS-A [20] that is presented to the participants in a playful format.

The program was developed under the metaphor of an enchanted forest in which happiness was kidnapped; each ability was presented in a didactic way with names of “powers” to obtain to overcome the game. In addition, the program was developed with an intermittent and variable reinforcement system that was represented through the gain of “power diamonds” by showing expected behaviors based on DBT skills. Additionally, there were weekly practice activities, and a character was included to guide students in using skills in their life situations.

**Figure 1:**
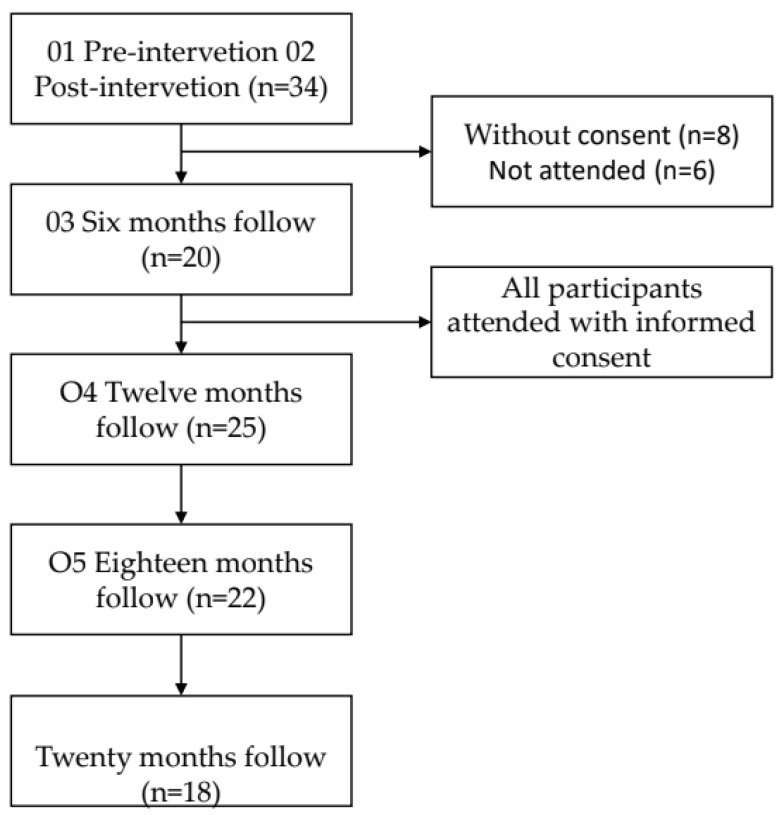
The flow of participants showers in the periods in which the follow-up evaluations of suicide risk behaviors, emotional dysregulation, depression, and psychological resources were assessed.

The program, previously piloted, consisted of the implementation of 25 sessions (Figure 1) corresponding to five modules structured and interconnected through metaphors, in which the set of DBT skills (Mindfulness, Discomfort Tolerance, Emotional Regulation, Interpersonal Effectiveness) and a module was added to work on self-knowledge and self-assessment.

Each session lasted 120 min and was implemented by psychology professionals trained in DBT. The program followed the structure of the DBT skills training sessions; a mindfulness activity followed by a homework review, a demonstration of a new skill, a homework assignment, and a session close. The program was applied by psychology professionals trained in DBT.

The present study was implemented in two stages. In stage 1, the school was asked for information to contact the parents and adolescents chosen for the study personally; but this was not possible. Then, only the parents and adolescents who participated in the intervention group were contacted. They were made aware of the purpose of the study, the potential risks, and the benefits. Informed consent was obtained from the parents, and informed assent from the adolescents.

Subsequently, stage 2 was implemented, where follow-ups were carried out 6, 12, 18, and 24 months after completing the DBT-PAHSE. The first follow-up (6 months) was carried out on 18 and 19 January 2020 through face-to-face interviews that the members of the research team carried out at the psychological care and training facilities of a sponsoring company in the town; Likewise, some interviews did at the home of the participants, at the express request of the parents.

The second follow-up (12 months) was from 20 to 30 July 2020. Given the conditions of social distancing due to the COVID-19 pandemic, the FICVIDA was in a digitized version through the Google platform. The test was applied by the research team members previously trained for its proper use. The FICVIDA was carried out via video call through the Google Meets platform. The evaluator presented the instrument to the participant and read each question and response option. The interviewer recorded the response indicated by the participant on the platform.

After the integration and analysis of the data, the results were returned to the parents with observations and suggestions derived from the evaluation. Additionally, parents were included in a social network to which they were sent information on validation, establishing rules and limits, and general guidance on adolescent development. It should be noted that the participants at risk of suicide due to attempts and ideation were referred to psychological treatment.

The third follow-up (18 months) was carried out from 20 January to 23 February 2021, as mentioned above. Finally, the fourth follow-up (24 months) was carried out from 23 July to 9 August 2021, with the same procedure established by the pandemic.

### 2.4. Data Analysis

In addition to the descriptive analysis of central tendency parameters, a model was used general linear of repeated measures, using a full factorial model (sum of squares type III) with simple contrasts to evaluate the differences in the variables associated with the risk of death from suicide for two years. Previously, the normalization of the data using the logarithmic transformation of the variables. Where included the pre-treatment (O1) and post-treatment (O2) measures and the four follow-ups during the 24 months after the implementation of the DBT-PAHSE (O3 = six months, O4 = twelve months, O5 = eighteen months, O6 = twenty-four months). The intra-subject factor was defined as the time observed at the end of the treatment, with 6 six levels and five levels for the case of Psychological Resources that were not evaluated in the six-month follow-up.

The intra-subject variables introduced to define this factor were: (a) suicide attempt; (b) suicidal ideation; (c) emotional dysregulation; (d) depression; (e) psychological resources; (f) affective psychological resources (management of sadness, anger management, self-control, recovery of balance); (f) resources social psychological (support network, asking for support, altruism); (h) psychological and cognitive resources. For the execution of the respective analyzes, statistical analysis software was used, SPSS 25.

## 3. Results

Eighteen of the total participants included in the study completed all the evaluations scheduled in the established period. Figure 1 shows the flow of participants included in each observation.

General linear analysis of repeated measures revealed statistically significant differences in reducing emotional dysregulation, with effect sizes very close to high and a statistical power close to 0.80 (F = 2.36; *p* = < 0.05; η² = 0.12; β = 0.72). The post hoc analysis carried out with Bonferroni did not indicate significant differences in the increase in the mean at twelve and eighteen months. The values of means and standard deviations in each observation are in detail in Table 1. Regarding suicidal behavior and its associated risk factors, Table 1 shows that suicidal ideation showed statistically significant differences in the period studied.

The post hoc analysis with the Bonferroni test showed that these differences were mainly explained by reduced suicidal ideation after six months and eighteen months at 24-month follow-up. However, there was also a significant increase from six to eighteen months of follow-up. Suicide attempts showed no change statistically significant (F = 1.14; *p* = 0.30).

**Figure 2:**
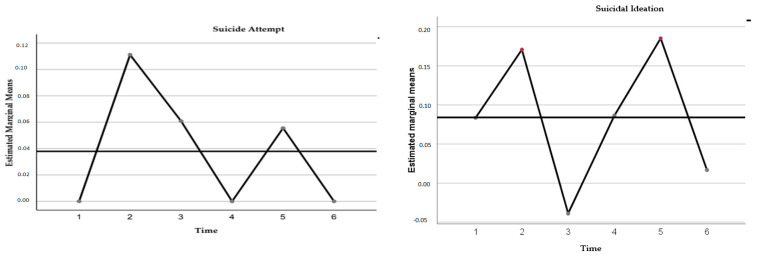
Trajectories of ideation and self-injuries behavior were evaluated in 18 early-onset adolescents who underwent intervention with the DBT-PAHSE program. The times correspond to the following periods: 1 = Pre-treatment evaluation (August 2018), 2 = Post-treatment evaluation (July 2019), 3 = Six-month follow-ups (January 2020), 4 = Twelve-month follow-ups (July 2020), 5 = 18-month follow-ups (January 2021), 6 = 24-month follow-up (July 2021).

Regarding malaise and elevated depressive symptoms, differences were also observed as statistically significant over time. High depressive distress noted a reduction from pre to post-evaluation (Mpre = 1.28 IC = 1.09–1.48; Mpost = 0.17 IC = 0.01–0.32) and, in the evaluation from eighteen to twenty-four months (M18 = 1.39 CI = 1.23–1.55; M24 = 1.32 CI = 1.16–1.49); likewise, it significantly increased at six, twelve, and eighteen months (Figure 2).

The intra-subject effects on each of the emotional dysregulation factors were analyzed. As shown in Table 2, none of the four evaluated showed significant changes in the observed time, even though the values of the means reflected decreased throughout the evaluations.

Table 3 shows that the psychological resources generally showed increases in the mean; however, these were not statistically significant (F = 2.16; *p* = 0.11; η² = 0.11; β = 0.60). Table 3 shows statistically significant differences over time for affective and social resources. Affective Resources registered mean increases in post-evaluations (M = 0.50 IC = 0.46–0.53), twelve-month follow-up (M = 0.51 IC = 0.49–0.53), maintenance at 18-month follow-up, and, finally, a decrease in the follow-up at twenty-four months (M = 0.40 CI = 0.50–0.55). Social resources reflected increases in their means from the post-evaluation to the eighteen-month follow-up; however, at the 24-year follow-up, a decrease in the mean value was recorded.

**Figure 3:**
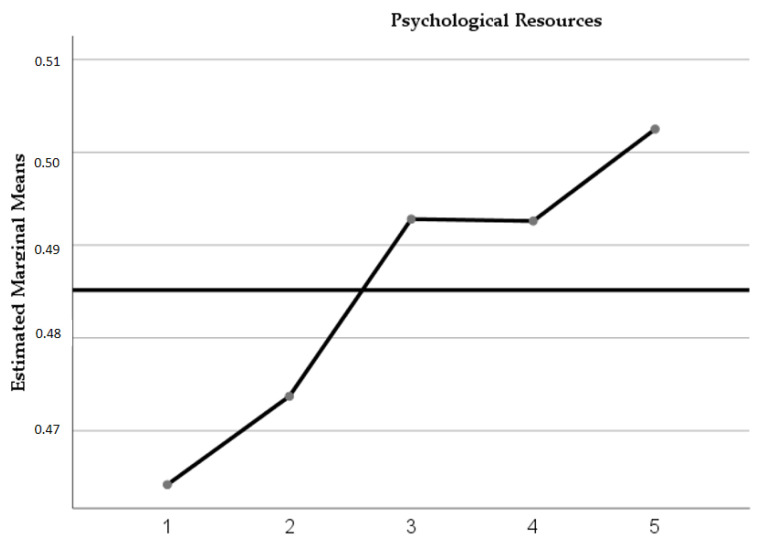
Trajectories of psychological resources were evaluated in 18 early-onset adolescents who were treated with the DBT-PAHSE program. The times correspond to the following periods: 1 = Pre-treatment evaluation (August 2018), 2 = Post-treatment evaluations (July 2019), 3 = Follow-up at six months (January 2020), 4 = follow-ups at twelve months (July 2020), 5 = follow-ups at 18 months (January 2021), 6 = 24-month follow-up (July 2021).

The dimensions of request help (F = 2.45; *p* = 0.05; η² = 0.12; β = 0.66) and altruism (F = 2.45; *p* = 0.05; η² = 0.12; β = 0.67), corresponding to social resources, also registered significant differences over time. Both resources showed increases in the mean values concerning the pre-evaluation. Altruism increased its mean at the follow-up at twelve and eighteen months, while asking for help maintained a mean above the post-evaluation during all the follow-up evaluations (Figure 3).

## 4. Discussion

The purpose of this study was to evaluate the effect of time on suicidal behavior and the proximal psychosocial factors of risk and protection associated with it. The researchers evaluated the maintenance of the changes achieved in a group of early-onset adolescents who participated in the DBT skills training program, DBT-PAHSE, adapted for Mexican school contexts. The changes analyzed were about suicide attempts, suicidal ideation, emotional dysregulation, depressive discomfort, affective psychological resources, and social psychological resources.

In general, the results obtained in this study reveal significant and differential changes in the variables studied after two years of follow-up. Additionally, it is highlighted that the most relevant changes occurred at twelve and eighteen months of follow-up when the adolescents were under conditions of social distancing due to the health emergency caused by COVID-19.

It is relevant that during the two years of follow-up, there were no cases of death by suicide nor any highly fatal suicide attempt. The absence of significant differences in the repeated measures analysis in these variables indicates that suicidal behavior remained stable and did not increase. This result provides relevant evidence regarding the scope that DBT-PAHSE can have in the prevention of deaths by suicide in the early-onset Mexican adolescent population; Likewise, it is promising for a country such as Mexico, where percentage increases of more than 400% in the rates of death by suicide in adolescents between 10 and 14 years of age have been reported over the last two decades [9]. On the contrary, suicidal ideation did show significant changes during follow-up (F = 2.55, *p* = 0.03). These changes occurred throughout the follow-up period without marking a significant difference in those made during the pandemic. Further analysis of how DBT skills and suicidal behavior are associated could help understand the permanence and functionality of suicidal ideation.

The most striking result concerns the time effects observed in emotional dysregulation and depressive symptomatology, referred to by the CESD authors as depressive distress. In both variables, statistically significant differences were found over time; however, the temporal change trajectories differed. While emotional dysregulation showed a generalized decrease at the end of the two-year follow-up (F = 2.36, *p* = 0.04) with high effect size and power (η^2^ = 0.12, β = 0.72), depressive symptoms increased (F = 53.02, *p* = 0.001), in the same way, with high effect size and power (η^2^ = 0.13, β = 1.0). It is important to note that the changes observed over time showed increases for both variables during follow-ups four and five, corresponding to the period in which the adolescents were under conditions of social distancing caused by the COVID-19 pandemic.

These findings raise important questions about the participation and efficacy of DBT skills in reducing emotional dysregulation and not reducing depressive distress. In this regard, McRae et al. [41] highlight the preponderant role of brain development. Their findings indicate that during early adolescence, it is difficult to find optimal maturation of those brain areas involved in re-evaluating negative affect and, therefore, depressive distress. This evidence implies that early adolescence is a critical development period to integrate effective emotion regulation strategies and that it is appropriate to intervene with this type of program due to the brain’s plasticity to learn them. In the same order of ideas, adolescence is a critical period to implement skills learning programs due they have more remarkable plasticity, understood as the flexibility or capacity necessary to modify their behavior and adjust to the demands of the environment [42,43]. In this sense, the evidence suggests that the transaction of learning DBT-PAHSE skills and brain development and plasticity favored the reduction of emotional dysregulation, even despite the increases observed during the pandemic. These results are consistent with those reported by Moore et al. [44,45,46].

On the other hand, it can be inferred that the increase in emotional dysregulation observed during the pandemic is caused by the interference caused by experiencing highly stressful life events in the use and effectiveness of learned skills. In this sense, it is essential to note that contextual variables influence the effective use of DBT skills [47]. Adolescents not only need to learn the skills but also learn to differentiate the right moment and time to implement them effectively. In this scenario, the skills learned by adolescents have been interfered with by increased emotional vulnerability caused by the transition from primary to secondary education and the social confinement caused by the pandemic. The change in the level of schooling implies a change in social and academic goals and adaptation to different contexts.

Regarding the increase in depressive discomfort, it drew particular attention because, based on what was proposed by Linehan [22] in the biosocial model and by Berking [48], it was expected that the reduction in emotional dysregulation would also imply a reduction in depression symptoms. The results obtained in this variable are similar to those reported in a study by Zapolski and Smith [27] with adolescents enrolled in middle school. These adolescents learned DBT skills with a brief program of nine sessions where the reduction of depressive symptoms was not observed; on the contrary, it also increased after a follow-up six months after the end of the program. On the other hand, there is evidence that depressive symptoms are reduced when adolescents are treated with standard DBT or comprehensive DBT programs [49,50].

The evidence noted seems to indicate that depression symptoms experienced in adolescence are hardly moderated by DBT skills learning when used as an independent intervention; however, it would be necessary to make some helpful contextual considerations to outline some hypotheses. Gonçalves et al. [51] point out that the relationship between depression and emotional dysregulation during adolescence can be explained by the lack of awareness of emotions that leads the adolescent not to know what needs to change and consequently be more easily exposed to persistent negative emotions. According to this position, a lack of awareness, clarity, and acceptance of emotions, particularly those experienced as unfavorable, may be related to increased depressive symptoms. In this sense, the results found in the present study did not show changes over time for difficulty in emotional awareness (F = 0.72, *p* = 0.60, η^2^ = 0.04, β = 0.25), difficulty in emotional clarity (F = 1.30, *p* = 0.28, η^2^ = 0.07, β = 0.18), difficulty in accepting emotions (F = 0.98, *p* = 0.43, η^2^ = 0.05, β = 0.33), nor in achieving goals (F = 1.38, *p* = 0.24, η^2^ = 0.07, β = 0.38). A subsequent study on DBT skills’ contribution to these variables could strengthen the program and make it more efficient in reducing depressive discomfort.

The results of the increase in depressive discomfort can also be read in the socio-health context derived from the COVID-19 pandemic. In this historical period, mental health problems such as anxiety and depression increased [52] in adolescents. The social dynamic was inserted into uncertainty, changes in routines, family confinement, job losses, and anxiety increased due to fear of the risk of contagion, increased deaths, and other changes that could increase health problems [52,53]. On the other hand, the pandemic experienced in follow-ups four and five increased the tension in family and school interactions. It is known that in children and early-onset adolescents, depression is associated with performance goals and is linked to the fundamental need to validate self-esteem. Emotional vulnerability at this stage of development is related to parenting styles characterized by extreme care, criticism, and disapproval that generate a feeling of uncertainty about one’s worth [54,55]. Under the above, it can be assumed that DBT skills are interfered with by a highly disabling context with essential stressors in achieving academic goals and interpersonal relationships with parents and caregivers.

Regarding the psychosocial protective factors associated with suicidal behavior, the significant effects of time on affective and social psychological resources provide evidence of the efficacy of DBT-PAHSE in acquiring and maintaining these resources. In this sense, the study’s results add to the evidence of various clinical trials that have shown the positive effect of training in DBT skills as an independent intervention both in clinical and school contexts [46,56]. Specifically, the DBT PAHSE program increased social resources (ability to request help and altruism) with similar results to those reported by Gasol [46]) after the implementation of DBT STEPS-A in Spanish adolescents. These results strengthen the initial evidence for the DBT PAHSE as a program for universal adolescent suicide prevention. The results reported by Rivera-Heredia and Andrade [40] indicate that a high level of affective resources is related to low levels of suicide risk.

Some limitations of the present study should be mentioned. First, it would be essential to consider carrying out future studies with follow-ups with the control group. Secondly, it is important to obtain data on the program’s effectiveness in the other genders of the adolescent population since the data obtained correspond only to the female gender. Third, it is desirable to replicate more studies that provide more significant evidence of the variables studied. Finally, the evaluation of the acquisition of DBT skills must be included, and analyses carried out on their possible mediating role. Authors should discuss the results and how they can be interpreted from the perspective of previous studies and of the working hypotheses. The findings and their implications should be discussed in the broadest context possible. Future research directions may also be highlighted.

## 5. Conclusions

In conclusion, the DBT-PAHSE program prevented and kept suicide deaths stable. It showed mixed results in reducing psychosocial risk factors. On the one hand, it favored the reduction of emotional dysregulation, and on the other hand, it failed to reduce depressive discomfort up to two years after treatment in adolescents. Then, the program requires improvements to reduce depressive discomfort, especially a variable strongly associated with suicide risk.

The program facilitated an increase in effective and social resources over time. However, a reinforcement session is required six months or a year after completion to avoid decreasing these resources.

DBT-PAHSE seems promising to reduce deaths by suicide and the emotional dysregulation that the developmental process can imply with preventive effects over time in Mexican adolescents. However, more studies are required to generalize the results and improvements to reduce depressive symptoms over time.

## Figures and Tables

**Table 1:** Intrasubject effects of adolescents who underwent DBT-PAHSE. Follow-up at 6, 12, 18 and 24 months (n = 18).

Study Variables	O1		O2		O3		O4		O5		O6					
	M	SD	M	SD	M	SD	M	SD	M	SD	M	SD	F	*p*	η^2^	β
Suicide Attempt	0.00	0.00	0.11	0.32	0.06	0.23	0.00	0.00	0.06	0.23	0.00	0.00	1.14	0.3	0.06	0.25
Suicidal Ideation	0.08	0.20	0.17	0.31	−0.03	0.23	0.08	0.27	0.18	0.35	0.01	0.07	2.55	0.03	0.13	0.77
Emotional Dysregulation *	1.74	0.15	1.68	0.13	1.64	0.13	1.68	0.13	1.67	0.13	1.62	0.11	2.36	0.04	0.12	0.72
Depression	1.28	0.38	0.17	0.31	1.31	0.32	1.36	0.31	1.4	0.32	1.32	0.33	53	<0.001	0.77	1

* Correction with Greenhouse-Geiser (*p* = 0.67) since the Mauchly sphericity test yielded *p* = 0.41.

**Table 2:** Intra-subject effects on the sub-dimensions of Emotional Dysregulation (n = 18).

Study Variables	O1		O2		O3		O4		O5		O6					
	M	SD	M	SD	M	SD	M	SD	M	SD	M	SD	F	*p*	η^2^	β
Emotional Dysregulation *	1.74	0.15	1.68	0.13	1.64	0.13	1.68	0.13	1.67	0.13	1.62	0.11	2.36	0.04	0.12	0.72
Not accept emotions	0.88	0.26	0.84	0.20	0.77	0.13	0.83	0.16	0.81	0.16	0.78	0.18	0.98	0.43	0.05	0.33
Difficulty achieving goals *	0.95	0.23	0.86	0.15	0.88	0.16	0.83	0.16	0.90	0.17	0.84	0.14	1.38	0.24	0.07	0.38
Diff. Emotional Awareness	0.77	0.22	0.81	0.19	0.76	0.20	0.83	0.19	0.84	0.20	0.79	0.13	0.72	0.60	0.04	0.25
Diff. Emotional Clarity *	1.08	0.16	1.03	0.16	0.98	0.18	1.02	0.14	1.00	0.12	0.98	0.12	1.30	0.28	0.07	0.18

* Correction with Greenhouse-Geiser (*p* = 0.67) since the Mauchly sphericity test yielded *p* = 0.41.

**Table 3:** Intra-subject effects on Psychological Resources and their dimensions (n = 18).

Study Variables	O1		O2		O3		O4		O5		O6					
	M	SD	M	SD	M	SD	M	SD	M	SD	M	SD	F	*p*	η^2^	β
Psychological Resources *	0.46	0.05	0.47	0.07	--	--	0.49	0.05	0.49	0.03	0.50	0.05	2.16	0.11	0.11	0.49
Affective Resources	0.44	0.06	0.50	0.08	--	--	0.51	0.06	0.47	0.06	0.40	0.05	3.94	0.01	0.18	0.82
Social Resources	0.47	0.06	0.49	0.07	--	--	0.51	0.04	0.52	0.03	0.52	0.04	3.50	0.01	0.17	0.80
Cognitive Resources	0.43	0.22	0.43	0.15	--	--	0.46	0.18	0.49	0.06	0.47	0.15	0.41	0.79	0.02	0.14
Sadness Management *	0.40	0.14	0.45	0.10	--	--	0.42	0.12	0.46	0.07	0.47	0.07	1.51	0.22	0.08	0.37
Anger Management +	0.44	0.10	0.42	0.10	--	--	0.49	0.06	0.46	0.08	0.48	0.09	2.09	0.09	0.11	0.60
Self-Control	0.45	0.15	0.45	0.14	--	--	0.48	0.12	0.45	0.09	0.51	0.05	1.12	0.35	0.06	0.33
Balance Recovery	0.44	0.08	0.46	0.10	--	--	0.45	0.11	0.47	0.11	0.44	0.11	0.34	0.84	0.02	0.12
Support Networks	0.48	0.16	0.50	0.16	--	--	0.52	0.08	0.52	0.05	0.52	0.08	0.86	0.49	0.04	0.26
Ask For Help +	0.38	0.15	0.45	0.09	--	--	0.44	0.07	0.43	0.08	0.47	0.08	2.45	0.05	0.12	0.66
Altruism	0.51	0.14	0.51	0.16	--	--	0.55	0.07	0.57	0.03	0.56	0.04	2.45	0.05	0.12	0.67

* Greenhouse-Geiser Correction. + Huynh-Feldt Correction.

## Data Availability

The study was conducted in accordance with the Declaration of Helsinki and approved by the Autonomous University of Aguascalientes Bioethics Committee with the protocol code CIB-UAA-23 approved of October 8th, 2019.
